# Iatrogenic retinal detachment secondary to inadvertent subretinal injection during posterior sub-Tenon triamcinolone injection

**DOI:** 10.3205/oc000094

**Published:** 2019-02-12

**Authors:** Rajya Laxmi Gurung

**Affiliations:** 1 Vitreo-retina Department, Biratnagar Eye Hospital, Biratnagar, Nepal

**Keywords:** retinal detachment, triamcinolone acetonide, cystoid macular oedema, pars plana vitrectomy

## Abstract

**Objective:** To report a case of iatrogenic retinal detachment due to inadvertent globe penetration during posterior sub-Tenon injection

**Methods:** A 65-year-old female was given posterior sub-Tenon injection of triamcinolone acetonide for pseudophakic cystoid macular oedema. The globe was inadvertently perforated with superior macula off retinal detachment.

**Results:** She underwent immediate pars plana vitrectomy but failed to regain significant vision improvement.

**Conclusion:** According to the literature, retinal detachment as a complication of posterior sub-Tenon injection is rare. This complication may be prevented by adhering to the standard practice of injecting slowly avoiding any undue pressure.

## Introduction

Posterior sub-Tenon (PST) injection of triamcinolone is commonly used in uveitic conditions, diabetic macular oedema, and macular oedema secondary to retinal vein occlusions and cystoid macular oedema. First described by Nozik in 1972 [1], PST injection is considered a relatively safe procedure. However, these techniques are not exempt from complications. A number of complications can occur including an increase in IOP, cataract, pseudoptosis, retinal and choroidal vascular occlusions, strabismus, and inadvertent globe penetration. Inadvertent globe penetration is a rare but sight-threatening complication. There are very few reported cases of this condition in literature. We report a case of iatrogenic retinal detachment secondary to inadvertent sub-retinal injection of triamcinolone acetonide (TA).

## Case description

A 65-year-old female was referred to our department with pseudophakic cystoid macular oedema (CME) in her left eye (LE). According to her records, she was initially given topical non-steroidal anti-inflammatory drugs followed by steroid drops with no significant improvement in vision. 

On examination, the best corrected visual acuity (BCVA) in the LE was 20/120. Fundus evaluation and OCT revealed pseudophakic CME. The decision was made to give PST in LE. After topical anaesthesia, 0.5 ml (Kenalog 40 mg/ml in 1 ml) was injected on the superotemporal quadrant with a 27G needle by a trainee. 

Immediately after the procedure in the left eye, inadvertent globe penetration was detected via indirect signs: red reflex became white, severe shallowing of the anterior chamber and immediate profound loss of vision, and a soft eye on digital palpation. 

**Figure 1:**
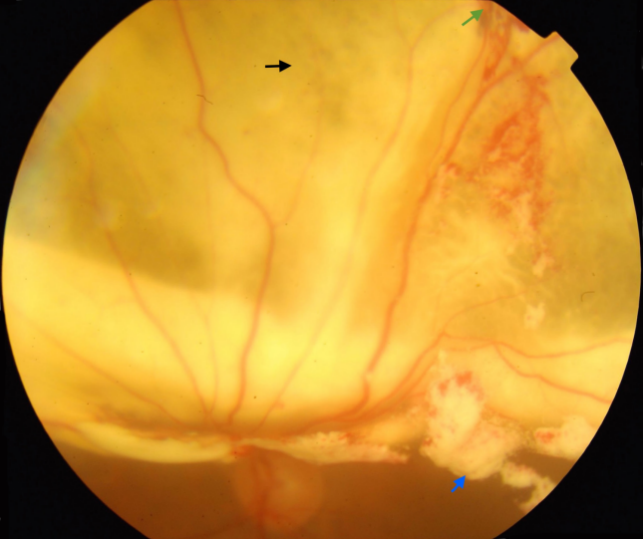
Figure 1 : Macula off superior retinal detachment showing areas of patch retinal necrosis (black arrow), site of globe perforation (green arrow), and free triamcinolone particles (blue arrow) in vitreous cavity

On examination, left eye fundus evaluation revealed sub-retinal triamcinolone with patchy retinal necrosis with macula off superior retinal detachment (Figure 1 (Fig. 1)). 

**Figure 2:**
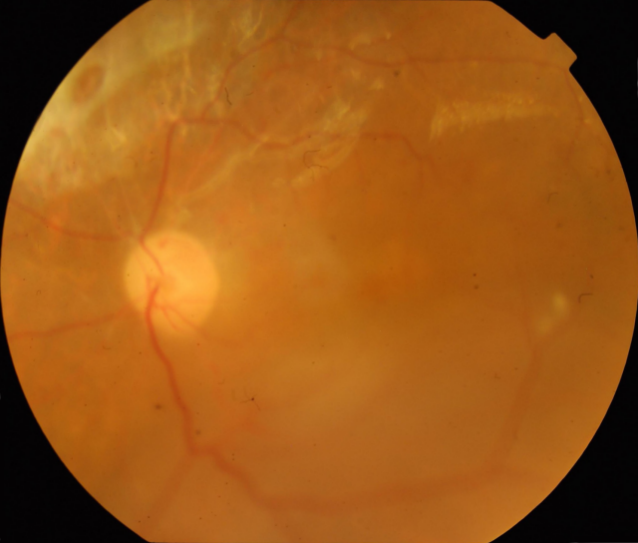
Attached retina under silicon oil

The patient underwent immediate pars-plana vitrectomy (PPV) with removal of the subretinal triamcinolone along with silicon oil insertion. During vitrectomy, 1 retinal tear associated with retinal detachment was noted on the superior retina along with subretinal particles of triamcinolone over the macular area. Some triamcinolone particles were also found dispersed in the vitreous cavity. Exploration of the surgical site of the injection was done, with a puncture site detected posterior to pars plana, corresponding to the location of the insertion of the needle. However, the patient developed re-detachment at 3-month follow-up for which she underwent repeat PPV. At 6-month follow-up, the retina was attached under oil with vision of HM (Figure 2 (Fig. 2)).

## Discussion

Though PSTI is fairly safe, a sub-Tenon injection is more difficult to perform than other periocular injection techniques. To achieve an accurate approach to the sub-Tenon space, the needle has to be placed close to the sclera. This possibly increases the incidence of ocular penetration. There are very few reported cases of globe perforation with retinal detachment during sub-Tenon injection. In their retrospective review, Kuo et al. reported a case of globe perforation out of 64 injections yielding an incidence of 1.56% [2]. Retinal photocoagulation was immediately performed around the penetration site with no further sequelae. Giny et al. reported a case of globe penetration during sub-Tenon injection pericataract surgery resulting in macula on supero-temporal retinal detachment which was managed with pars-plana vitrectomy, however the final visual acuity was 6/6 with no long-term sequeale [3]. Gomez-Ulla et al. also reported a case of unintentional ocular injection of TA during sub-Tenon injection [4]. The case was managed conservatively with full gain of vision. Surgical intervention in our case was believed to be necessary to address the macula off retinal detachment. Despite immediate pars plana vitrectomy, the final visual outcome was still poor in our case. However, all globe penetrations are different, and an assessment of globe anatomy, visual acuity, and fundus evaluation should guide whether surgical or conservative management is most appropriate.

In this particular case, the procedure was done by a junior resident doctor, suggesting the importance that the technique be performed by an experienced physician or under strict supervision. State of the art is using an Atkinson retrobulbar needle which reduces the risk of inadvertent ocular penetration significantly. However, Atkinson needle is not availabe in our setup and 27/26 G needle is routinely used. Moreover, one has to inject very slowly keeping in mind the features of inadvertent globe penetration which might be missed. Kuo et al. [2] also reported inadvertent globe penetration by a junior resident doctor in their series, suggesting the importance that the technique be performed by an experienced operator.

TA is known to be toxic to the retina and retinal pigment epithelium, particularly at high doses. This was confirmed in our case, where 20 mg (0.5 mL) subretinal triamcinolone resulted in patchy areas of retinal necrosis (Figure 1 (Fig. 1)). Despite attached retina, there was no significant improvement in visual acuity probably due to triamcinolone induced retinal toxicity.

## Conclusion

We report this case to make ophthalmologists aware of this vision-threatening complication of posterior sub-Tenon injection of steroids. One should adhere to the standard practice of injecting slowly to avoid excessively forceful injection and to stop the injection as soon as any undue pressure is felt during injection.

## Notes

### Competing interests

The author declares that she has no competing interests.
